# Enhanced serum immunoglobulin G clearance in myotonic dystrophy-associated hypogammaglobulinemia: a case series and review of the literature

**DOI:** 10.1186/s13256-019-2285-3

**Published:** 2019-11-20

**Authors:** Sarah C. Sasson, Alastair Corbett, Andrew J. McLachlan, R. Chen, S. A. Adelstein, Sean Riminton, Sandhya Limaye

**Affiliations:** 1 Nuffield Department of Medicine, Experimental Medicine Division, University of Oxford, Level 5, John Radcliffe Hospital, Oxford, OX3 9DU UK; 2 Department of Neurology, Concord Hospital, Sydney, Australia; 3 Sydney Pharmacy School, University of Sydney, Sydney, Australia; 4 Immunopathology Laboratory, Department of Clinical Immunology, Royal Prince Alfred Hospital, Sydney, Australia; 5 Sydney Medical School, University of Sydney, Sydney, Australia; 6 Department of Clinical Immunology, Concord Hospital, Sydney, Australia

**Keywords:** Case report, Myotonic dystrophy type 1, DM1, Hypogammaglobulinemia, IgG, Intravenous immunoglobulin, IVIg

## Abstract

**Background:**

Myotonic dystrophy type 1 is an autosomal dominant disorder characterized by muscle weakness, myotonia, cataracts, and cardiac conduction defects; it is associated with expansions of cytosine-thymine-guanine repeats in the myotonic dystrophy protein kinase. Hypogammaglobulinemia is a lesser known association of myotonic dystrophy type 1 and the underlying pathogenesis of immunoglobulin G depletion remains unclear.

**Case presentation:**

Here we report a kindred of two members (a 62-year-old white woman and a 30-year-old white man; mother and son) with myotonic dystrophy type 1-associated hypogammaglobulinemia associated with altered intravenous immunoglobulin elimination kinetics and reduced half-life. There was no history of systemic immunosuppression or renal or gastrointestinal protein loss in either patient, and no underlying case for a secondary immunodeficiency could be found. One patient required fortnightly intravenous immunoglobulin to maintain adequate trough immunoglobulin G levels.

**Conclusions:**

Ongoing study of myotonic dystrophy type 1-associated hypogammaglobulinemia using contemporary tools of genomic medicine may help to further delineate the pathogenesis of this entity.

## Background

Myotonic dystrophy type 1 (DM1) is an autosomal dominant disorder characterized by muscle weakness, myotonia, cataracts, and cardiac conduction defects; it is associated with expansions of cytosine-thymine-guanine (CTG) repeats in the 3′ untranslated region of the myotonic dystrophy protein kinase (DMPK) on chromosome 19 (reviewed in [1]). Expansion of the CTG repeats is thought to underlie the pathogenesis of the main clinical features of DM1 and is mediated at the level of deoxyribonucleic acid (DNA) and ribonucleic acid (RNA) via mutant transcripts containing expanded cytosine-uracil-guanine (CUG) repeats. These repeats bind the regulator muscleblind-like 1 resulting in aberrant protein splicing.

The disease displays maternal transmission bias and the number of CTG repeats correlates positively with disease severity and inversely with the age of onset. Hypogammaglobulinemia is a lesser known association of DM1, first reported in 1956 [2]. While an early theory hypothesized that malfunction of the neonatal Fc receptor (FcRn) leads to hypercatabolism of immunoglobulin G (IgG), in fact the underlying pathogenesis of IgG depletion remains unclear. Here we report a kindred of two members with DM1-associated hypogammaglobulinemia associated with altered intravenous immunoglobulin (IVIg) elimination kinetics and reduced half-life (t_1/2_). One patient required fortnightly IVIg to maintain adequate trough IgG levels.

## Case presentation

### Case 1

**Fig. 1:**
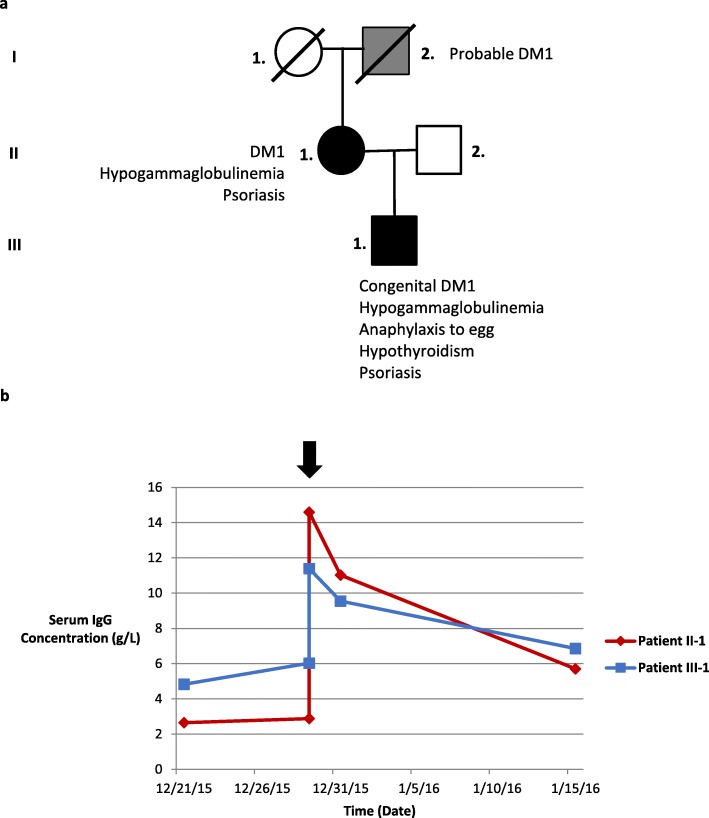
**a** Pedigree displaying two confirmed cases of myotonic dystrophy type 1-associated hypogammaglobulinemia (*black*). **b** Serum immunoglobulin G level displayed after intravenous immunoglobulin administration. First dose of intravenous immunoglobulin indicated by *arrow*. *DM1* myotonic dystrophy type 1, *IgG* immunoglobulin G

A 62-year-old white woman (Patient II-1 in Fig. 1a) with DM1 was investigated after routine testing in a specialist DM1 clinic found the serum IgG concentration was 2.78 g/L (reference range 7.0–16.0 g/L). IgG concentration a year prior had also been low at 4.0 g/L. There was no recent history of recurrent sinopulmonary, gastrointestinal, or unusual infections.

Her past medical history included recurrent upper respiratory tract infections during childhood with adenoidectomy and tonsillectomy. Her DM1 symptoms were recognized only after the birth of her son (Patient III-1 in Fig. 1a), when he was diagnosed as having congenital DM1. Her past medical history also included psoriasis that was never treated with systemic immunosuppression. In regard to family history, she believes her deceased father also displayed ptosis and distal weakness; however, he was never formally diagnosed as having DM1. In regard to social history, she was born in Ireland and she did not smoke tobacco or drink alcohol. There were no pets at home. She worked part-time in a small business and there were no discernible environmental exposures. Patient II-1’s medications were: conjugated estrogen hormone replacement therapy, ezetimibe 10 mg daily, cholecalciferol 1000 IU daily, and acitretin 10 mg daily. The clinical features of Patient II-1 are summarized in Table 1.

**Table 1:** Clinical features of Patient II-1 and III-1

Patient	Demographics	Presenting complaint	Past medical history	Interventions for infectious illnesses to date
Patient II-1	62 F	Asymptomatic	DM1 Recurrent tonsillitis in childhood Psoriasis	Adenoidectomy Tonsillectomy
Patient III-1	30 M	Recurrent small bowel obstructions	Congenital DM1 Recurrent otitis media Recurrent small bowel obstructions Hypothyroidism Anaphylaxis to egg Psoriasis	Bilateral grommet insertion Medical management of small bowel obstructions

*DM1* myotonic dystrophy type 1, *F* female, *M* male

A neurological examination found facial weakness, ptosis, handgrip and percussion myotonia, distal wasting, and weakness of her upper and lower extremities with bilateral foot drop requiring a walking cane. There were psoriatic plaques on her hands and forearms bilaterally. There was no active synovitis or palpable lymphadenopathy. Her mouth was clear. Her heart rate was 60 beats per minute and regular. Her heart sounds were dual with no murmur. Her chest was clear to auscultation. Her abdomen was soft and non-tender and there was no peripheral edema.

Laboratory investigations revealed unremarkable full blood count (FBC), electrolytes, urea, and creatinine (EUC), and liver function tests (LFT) (Table 2). The serum albumin was within normal limits. There was no evidence of urinary protein loss (Table 2). Further investigations into secondary causes of hypogammaglobulinemia found no abnormal lymphocyte populations by flow cytometry with normal proportion of switched memory B cells (Table 3). Serology testing showed protective levels of IgG to diphtheria, *Haemophilus influenzae*, and tetanus, and low to pneumococcus (Table 3). Serum and urine protein electrophoresis confirmed hypogammaglobulinemia without evidence of significant proteinuria and immunofixation did not detect a paraprotein.

**Table 2:** Laboratory findings in Patient II-1 and Patient III-1

	Patient II-1	Patient III-1	Reference range
White cell count (cells/μL)	5.0	**3.9**	4.0–10.0
Hemoglobin (g/L)	130	135	120–150
Platelets (cells/μL)	199	178	150–400
Sodium (mmol/L)	143	149	135–145
Potassium (mmol/L)	4.1	4.1	3.5–5.2
Chloride (mmol/L)	108	**111**	95–110
Bicarbonate (mmol/L)	22	25	22–32
Urea (mmol/L)	**8.9**	4.0	3.0–8.0
Creatinine (μmol/L)	84	66	45–90
Bilirubin (μmol/L)	8	30	< 21
Albumin (g/L)	42	41	38–48
Protein (g/L)	67	70	60–80
ALP (U/L)	68	52	30–110
GGT (U/L)	**88**	21	< 35
ALT (U/L)	22	28	5–55
AST (U/L)	25	33	5–55
Urine Protein:Creatinine (mg/mmol)	5.4	7.2	< 12
Urine red blood cells (cells/mL)	< 10	< 10	< 10
Urine white blood cells (cells/mL)	**10–100**	< 10	< 10

*ALT* alanine transaminase, *ALP* alkaline phosphatase, *AST* aspartate aminotransferase, *GGT* gamma-glutamyl transpeptidase

Results displayed in bold indicate values outside the given reference range

**Table 3:** Lymphocyte, memory B-cell populations, and serology

a)			
	Patient II-1	Patient III-1	Reference range
Lymphocyte count (cells × 10^9^/L)	1.9	**1.1**	1.2–2.7
CD3^+^ (%)	**86**	78	49–84
CD3^+^ (cells × 10^9^/L)	1.63	0.86	0.6–2.99
CD19^+^ (%)	**6**	10	7–27
CD19^+^ (cells × 10^9^/L)	0.11	0.11	0.11–0.7
CD4^+^ (%)	43	55	28–63
CD4^+^ (cells × 10^9^/L)	0.82	0.6	0.44–2.16
CD8^+^ (%)	40	20	10–40
CD8^+^ (cells × 10^9^/L)	0.76	0.22	0.13–1.31
CD16^+^CD56^+^ (%)	7	11	4–25
CD16^+^CD56^+^ (cells × 10^9^/L)	0.13	0.12	0.1–0.64
CD4^+^:CD8^+^ ratio	1.08	2.75	0.6–5
b)			
	Patient II-1	Patient III-1	Reference range^a^
Total CD19^+^ B cells (% of lymphocytes)	**4.9**	7.8	5–26
Total CD19^+^ B cells (cells × 10^9^/L)	0.093	0.086	58–558
CD19^+^ 27^+^ memory B cells (% of B cells)	41	11	7–48
CD19^+^ 27^+^ memory B cells (cells × 10^9^/L)	0.038	**0.0097**	0.013–0.148
CD19^+^ 27^+^IgM^−^IgD^−^ switched memory B cells (% of B cells)	**35**	5.8	3–23
CD19^+^ 27^+^IgM^−^IgD^−^switched memory B cells (cells × 10^9^/L)	0.032	0.0050	0.004–0.066
CD19^+^ 27^−^IgM^+^IgD^+^naïve B cells (% of B cells)	55	87	29–93
c)			
	Patient II-1	Patient III-1	Reference range
Diphtheria IgG (IU/mL)	0.26	n.d.	> 0.1
*Haemophilus influenzae* IgG	0.47	n.d.	N/A
Pneumococcal IgG (mcg/mL)	**7.5**	**16.9**	> 39
Tetanus IgG (IU/mL)	2.59	0.84	> 0.16

*IgG* immunoglobulin G. ^**a**^Based on published data. Abnormal results are shown in bold

Patient II-1 was commenced on 36 g Intragam® P (CSL, Behring, Australia) IVIg (that is, 0.4 mg/kg) monthly due to significantly low IgG. We performed *in vivo* pharmacokinetic (pK) investigations by measuring serial serum IgG at 1 hour, 48 hours, and 14 days after the initial infusions (Fig. 1b). The estimated elimination t_1/2_ of IgG was 12.7 days for Patient II-1 which was markedly reduced when compared to the reported t_1/2_ of IgG after Intragam P administration of 39.7 (+/− 7.8) days [3].

Over the following 6 months, Patient II-1 remained well and received 4-weekly IVIg. Monitoring of pre-infusion serum IgG levels revealed that Patient II-1’s serum IgG remained low at 5.0 g/L despite standard dose and timing. Increasing Patient II-1’s IVIg dosing to 21 g fortnightly increased her trough IgG to 7.8 g/L which is within normal limits. At the time of writing, Patient II-1 was in a stable condition.

### Case 2

The son of the index patient (Patient III-1 in Fig. 1a) is a 30-year-old white man with congenital DM1 who was also found to have a low IgG level of 5.34 g/L after routine testing in a specialist DM1 clinic.

The obstetric history of Patient II-1 was uncomplicated until premature labor occurred at week 32 of gestation. Patient III-1 was critically unwell at birth and spent several weeks in a neonatal intensive care unit. He was diagnosed as having congenital DM1 after genetic testing as an infant, which prompted genetic testing and diagnosis in the mother. He had recurrent otitis media as a child requiring bilateral grommet insertion at age 4 and had ongoing recurrent small bowel obstruction (with no identified pathogens), all consistent with an underlying antibody deficit. His past medical history also included psoriasis never treated with systemic immunosuppression, hypothyroidism, anaphylaxis to egg, and bilateral intraocular lens replacement. Patient III-1’s medications were: thyroxine 25 mcg daily, fish oil 1 tablet daily, cholecalciferol 1000 IU daily, and an epinephrine autoinjector when needed.

A family history confirmed Patient III-1’s father does not have DM1, and his maternal grandfather had probable DM1 as described above. In regard to social history, Patient III-1 lived with both parents. He worked part-time in a supervised workplace. There were no pets in the home and no discernible environmental exposures. The clinical features of Patient III-1 are summarized in Table 1.

A neurological examination found Patient III-1 had facial weakness, myotonia, distal upper and lower limb muscle wasting and weakness, and developmental delay. There was a singular psoriatic plaque on his right lower limb. There was no active synovitis or palpable lymphadenopathy. His heart rate was 60 beats per minute and regular. His heart sounds were dual with no murmur. His chest was clear to auscultation. His abdomen was soft and non-tender. There was no peripheral edema.

Laboratory investigations found FBC, EUC, and LFT were unremarkable. The serum albumin was within normal limits. There was no evidence of urinary protein loss (Table 2). Further investigations into secondary causes of hypogammaglobulinemia found no abnormal lymphocyte populations by flow cytometry with normal proportion of switched memory B cells (Table 3). Serology testing showed protective levels of IgG to tetanus and low to pneumococcus (Table 3). Serum and urine protein electrophoresis confirmed hypogammaglobulinemia without evidence of significant proteinuria and immunofixation did not detect a paraprotein.

Patient III-1 was commenced on 21 g Intragam P IVIg (that is, 0.4 mg/kg) monthly, due to low IgG and a history of recurrent infections (Patient III-1). We performed *in vivo* pK investigations by measuring serial serum IgG at 1 hour, 48 hours, and 14 days after the initial infusions (Fig. 1b). The estimated elimination t_1/2_ of IgG was 25.1 days for Patient III-1, markedly reduced when compared to the reported t_1/2_ of IgG after Intragam P administration of 39.7 (+/− 7.8) days [3].

Over the following 6 months Patient III-1 remained well with a trough IgG concentration of 6.44 g/L on 4-weekly dosing which is just above the lower limit of normal. At the time of writing, he had not been treated for a significant infection, and specifically no small bowel obstructions, since the commencement of IVIg.

## Discussion

Despite being first described 60 years ago, the mechanism of DM1-associated hypogammaglobulinemia remains poorly understood and the diagnosis is probably under-recognized outside specialist DM1 clinics. Here we report an affected kindred of two members, both who were diagnosed after routine screening. Interestingly, while Patient II-1 had very low serum IgG, her burden of infection was milder than her son’s and included recurrent upper respiratory tract infections during childhood and related adenoidectomy and tonsillectomy. Patient III-1’s serum IgG was only mildly decreased and yet his infection burden was greater, with recurrent otitis media necessitating grommet insertion and ongoing recurrent small bowel obstructions. A clinical decision was made to commence both patients on IVIg replacement, allowing for first-dose pK studies that contributed further *in vivo* data that demonstrated the serum clearance of IgG displays enhanced kinetics in this condition. For the first time we outline practical challenges in the clinical management of patients with DM1-associated hypogammaglobulinemia, including the need to administer IVIg at decreased intervals to maintain serum IgG levels in Patient II-1.

Hypogammaglobulinemia in the setting of DM1 has now been reproducibly reported with an early study finding 15 of 19 (79%) patients with DM1 affected [4]. Kaminsky *et al.* [5] studied 52 patients with DM1 and categorized them according to clinical status (Category 1–5 where 1 = no clinical features and 5 = non-ambulant patients). The median serum IgG was lower than healthy controls in all DM1 groups, but there were no further differences between groups. However, others have found lower serum IgG associated with Category 5 patients [6]. Compared to healthy controls, patients with DM1 had lower total immunoglobulin, total IgG, IgG1 and IgG3. There were no differences in immunoglobulin A (IgA), immunoglobulin M (IgM), IgG2, or IgG4 [5]. Serum levels of IgG and IgG1 and total and CD8^+^ T cell counts inversely correlated with the absolute number of CTG repeats in some studies [5] but not others [6].

Other work has found a longer duration of DM1 is associated with lower serum IgG levels, both in cross-sectional analysis and in longitudinal study of individual patients with DM1 over time [7]. Suzumura *et al.* found no differences in broad lymphocyte populations or mitogen-driven proliferation [7], nor any differences in *in vitro* production of IgG between patients with DM1 and healthy controls. Bone marrow cellularity and plasma cell counts were also comparable [7]. Injection of radiolabeled ^125^-I-IgG led to more rapid clearance of the ^125^-I-IgG from day 2–4 in six patients with DM1 compared with seven healthy controls. There was no difference in the fractional catabolic rate or t_1/2_ over the remaining 2 weeks of the study [4, 7].

The proposal that the FcRn receptor is altered in DM1 leading to hypercatabolism of IgG, possibly due to its proximity to DMPK on chromosome 19, remains controversial in the literature. FcRn is a major histocompatibility complex (MHC)-1-related receptor with distinct binding sites for IgG and albumin. FcRn bind these proteins, preventing degradation and prolonging their t_1/2_ while also recycling them to the circulation. In addition, FcRn transports IgG across the placenta. In a murine model, defective FcRn results in an increased catabolic rate of serum IgG and reduced serum level and t_1/2_ [8].

Proponents of the theory that underlying alterations in FcRn are the cause for low IgG in DM1 have used mathematical modelling of kinetic analysis [9] to demonstrate that a normal number of FcRn with lowered affinity for IgG but normal affinity for albumin may explain why most patients with DM1 have low concentration of serum IgG and normal serum albumin [4], as was shown in both of our cases.

However, other data do not support the proposal that alterations in FcRn results in low IgG in DM1, including human studies where FcRn transcript number was not lower in muscle biopsies or peripheral blood lymphocytes in patients with DM1, and the FcRn transcript number did not correlate with CTG repeat number or serum IgG level [6]. In addition, the fact that FcRn binds IgG3 with lower affinity than IgG1, IgG2, IgG3, IgM, and IgA does not support the low IgG1 and IgG3 found in DM1 and relative preservation of other immunoglobulins [10].

Although DM1-associated hypogammaglobulinemia is recognized in the literature [2, 5–7], it probably remains an under-recognized phenomenon, especially outside DM1 specialty clinics. There is no published data on the relationship between serum IgG levels and infection risk to guide the management of IVIg replacement in patients with DM1.

Here, we report a kindred with two confirmed cases of DM1-associated hypogammaglobulinemia with high systemic clearance of IgG. We did not investigate whether there was associated deficit of B cell antibody production *in vitro*, given previous work in this area showed no difference between patients with DM1 and healthy controls [7].

All serum IgG levels were measured in the same diagnostic immunopathology laboratory on a BNII nephelometer (Siemens, Munich, Germany). The diagnostic reference range was 7.0–16 g/L. The laboratory runs regular internal quality control using two controls near the lower limit of normal (Control 1 and 2) and a high-range control (Control 3). The mean standard deviations in the most recent 6-month-period audit were 0.38, 0.25, and 1.45, respectively. The coefficients of variance during the same period were 5.59%, 4.38%, and 8.17%, respectively.

We opted to commence both patients on IVIg. In the case of Patient III-1 this decision was supported by the low serum IgG level together with a significant history of infection (recurrent otitis media and bowel obstruction) that was consistent with a humoral immunity deficit. The decision to treat Patient II-1 was less clear, as although she had the more profound serum IgG deficit, her history of infection was less significant. We were influenced by the management and guidelines of common variable immunodeficiency (CVID), the most prevalent immunodeficiency presenting in adulthood, where a serum IgG of 2.78 g/L would be considered moderately severe and generally acted upon (as reviewed in [11]). However, we acknowledge there is a paucity of evidence to guide commencement of IVIg in DM1-associated hypogammaglobulinemia. An interesting aspect of this case was that Patient II-1’s high clearance of serum IgG resulted in fortnightly IVIg replacement dosing to maintain adequate IgG trough concentrations. It will be important to monitor Patient III-1 over time to determine if his IgG deficiency worsens, as has been previously reported [7]. The presence of psoriasis in both patients also raises the possibility of more widespread immune dysregulation.

## Conclusions and future directions

We postulate that DM1-associated hypogammaglobulinemia has a genetic basis but it is not currently recognized by the International Union of Immunological Societies as an inborn error of immunity [12]. The current era of genomic medicine appears to provide unprecedented opportunity to further delineate the pathophysiology of this entity, for example by assessing differences between patients with DM1 with and without low IgG in terms of FcRn and IgG RNA and potential regulator or intermediaries [9] between FcRn and IgG. Such investigations in larger scale studies would be of value to this field.

## Data Availability

The datasets used during the current study are available from the corresponding author on reasonable request.

## Abbreviations

CTG: Cytosine-thymine-guanine
CUG: Cytosine-uracil-guanine
CVID: Common variable immunodeficiency
DM1: Myotonic dystrophy type 1
DMPK: Myotonic dystrophy protein kinase
DNA: Deoxyribonucleic acid
EUC: Electrolytes, urea, and creatinine
FBC: Full blood count
FcRn: Neonatal Fc receptor
IgA: Immunoglobulin A
IgG: Immunoglobulin G
IgM: Immunoglobulin M
IVIg: Intravenous immunoglobulin
LFT: Liver function tests
MHC: Major histocompatibility complex
pK: Pharmacokinetic
RNA: Ribonucleic acid
t_1/2_: Half-life
